# An Architecture for Measuring Joint Angles Using a Long Period Fiber Grating-Based Sensor

**DOI:** 10.3390/s141224483

**Published:** 2014-12-19

**Authors:** Carlos A. Perez-Ramirez, Dora L. Almanza-Ojeda, Jesus N. Guerrero-Tavares, Francisco J. Mendoza-Galindo, Julian M. Estudillo-Ayala, Mario A. Ibarra-Manzano

**Affiliations:** 1 Laboratorio de Procesamiento Digital de Señales, Departamento de Electrónica, DICIS, Universidad de Guanajuato, Carr. Salamanca-Valle de Santiago Km. 3.5 + 1.8 Km., Salamanca 36885, Mexico; E-Mails: ca.perezramirez@ugto.mx (C.A.P.-R.); fj.mendozagalindo@ugto.mx (F.J.M.-G.); 2 Departamento de Ingeniería Robótica, Universidad Politécnica de Guanajuato, Av. Universidad Norte SN, Comunidad Juan Alonso, Cortazar 38483, Mexico; E-Mail: luzdora@ieee.org; 3 Laboratorio de Optoelectrónica, Departamento de Electrónica, DICIS, Universidad de Guanajuato, Carr. Salamanca-Valle de Santiago Km. 3.5 + 1.8 Km., Salamanca 36885, Mexico; E-Mails: lnbmt@gmail.com (J.N.G.-T.); julian@ugto.mx (J.M.E.-A.)

**Keywords:** fiber-optics sensor, long-period fiber grating, Gaussian mixture model, recursive least-square estimator, finger movement, real-time system

## Abstract

The implementation of signal filters in a real-time form requires a tradeoff between computation resources and the system performance. Therefore, taking advantage of low lag response and the reduced consumption of resources, in this article, the Recursive Least Square (RLS) algorithm is used to filter a signal acquired from a fiber-optics-based sensor. In particular, a Long-Period Fiber Grating (LPFG) sensor is used to measure the bending movement of a finger. After that, the Gaussian Mixture Model (GMM) technique allows us to classify the corresponding finger position along the motion range. For these measures to help in the development of an autonomous robotic hand, the proposed technique can be straightforwardly implemented on real time platforms such as Field Programmable Gate Array (FPGA) or Digital Signal Processors (DSP). Different angle measurements of the finger's motion are carried out by the prototype and a detailed analysis of the system performance is presented.

## 1. Introduction

Over the past years, the use of fiber-optics-based sensors have increased [1] due to the fact they offer: (1) immunity to electromagnetic interference; (2) immunity to radio frequency interference; and (3) electrical isolation. Moreover, their use in applications for remote sensing and portable devices have been expanding because: (a) no electric cables are required for their operation [2]; (b) they can be easily multiplexed; (c) their size can be reduced [3]; and (d) they are non-intrusive [2]. Hence, this type of sensor offers excellent performance in real-life environments. Several applications have been proposed for measuring physical properties such as: pressure [4], temperature [5,6], acceleration [7], humidity and strain using temperature insensitive sensors [8,9], displacement [10]. Further, the aforementioned features make them suitable for biomedical and biomechanical applications [11] like heart beat and respiration monitoring [12], respiration movement detection [13], force applied on teeth during realistic orthodontic treatments [14] and motion detection in joints [15].

The most widely fiber-optics sensors known are interferometers, fiber Bragg grating (FBG), long-period fiber grating (LPFG), distributed and polarimetric. In particular, applications for LPFG sensors have been reported in fields such as measurements of ionic concentration [16], levels of a liquid and fluid-flow velocity [17], strain [18,19], humidity [20], viscosity [21], refraction index [22], and as biosensors [23]. In spite of the wide range of proposed applications, a LPFG-based sensor for joint movement detection has not been reported. In this study, a LPFG-based sensor is chosen because: (i) it can work well in high-temperature environments; and (ii) the production cost is very low, when the electric arc discharge fabrication technique is used. Due to the latter, the sensor can be manufactured with fiber-optics for communications by only using a fusion splicer machine [24]. In contrast, the fabrication of FBG sensors requires the use of special fibers, making them more expensive than the ones used in this article. Further, the spectral response of a LPFG-based sensor under bending applications behaves in two different ways: a shift in the central wavelength attenuation [25,26] or the split in two of each attenuation band, with the wavelength separation of each formed band changing with the curvature [27]. These effects cause a better inherent sensitivity for this application than their FBG counterparts [25,28].

In the recent years, several systems have been proposed to measure the joint movement. The sensors used in these can be classified into the following categories: (1) magnetic; (2) resistive; (3) capacitive; (4) Inertial Measure Unit (IMU); and (5) fiber-optics. For instance, in [29] a system to detect finger tapping movements is developed, with the aim of helping doctors to diagnose Parkinson's disease. This system uses a magnetic-based sensor to detect the movements. An application using accelerometers to detect finger gestures, using a microcontroller as the processor unit, is described in [30]. This device detects specific static finger movements recognizing up to six different gestures. A glove using coil-based sensors is reported in [31]. Another glove for measuring finger flexion using resistive sensors is reported in [32]. In general, devices that use the first four categories are more sensitive to electromagnetic interference; thus, it is necessary to explore the use of other sensors which are less sensitive to this type of interference. Due to their characteristics, the use of fiber-optics-based sensors in this type of applications is increasing. For instance, a glove for detecting hand posture and movement at the finger joint by using FBG-based sensors is explained in [33]. The movement-detection algorithm is based on measuring the reflected spectrum. In [15], a glove is developed to detect movement in the finger joints by using Hetero-Core Fiber-Optic as the sensing element. Its operation is based on the light loss, which is proportional to the joint bent. Although the inventors of the gloves show that their sensors can detect joint movements, the detection rates are not reported. Furthermore, they use a Personal Computer (PC) as the processor unit, leading to a slower system response. Hence, designing an algorithm that can be implemented in an online system would be desirable.

A fiber-optics sensor modulates the beam light into phase difference [34], amplitude and polarization, among others, according to different parameters such as wavelength variation, absorption or scattering of the optical signal [2]. These changes can be processed using a photodetector that converts the modulated beam light into an electrical signal; however, this signal needs to be filtered due to the noise generated by the photodetector. Several strategies have been proposed to filter the signal. For instance, in [35] the authors suggest a technique based on the Wavelet Transform (WT), using a computer as the processor unit, to filter the signal from a FBG-based sensor that measures temperature. The method proposed uses MATLAB functions to filter the signal, based on the algorithm called “Denoising with a given soft or hard threshold”. According to this algorithm, a heuristic formula is used to compute a threshold value that serves as the input parameter of the function *wdencmp*, which filters the signal. In [36], a comparison is performed between five algorithms, using a computer as the main processor, to filter a signal from an FBG-based sensor. The filtering techniques involved in the comparison process are: (1) moving average filter; (2) median filter; (3) Finite Impulse Response (FIR)-based 51th order filter using a Kaiser window; (4) Infinite Impulse Response (IIR)-based 2nd order filter using the Butterworth approximation; and (5) zero-phase filter. The best results are obtained using the probabilistic filters (e.g., median and the moving average). On the other hand, several architectures that implement some filtering techniques in real-time controllers have been proposed. For instance, a System-on-a-chip (SoC) design using a one-dimensional WT is proposed in [37] to filter the signal from a fiber-optics based gyroscope. This architecture consists in the Discrete Wavelet Transform (DWT) implementation using the Mallat algorithm [38]. The algorithm uses 1024 samples for every batch of data. The results of this implementation are noticeable, but it consumes 60% of the resources on the FPGA (Virtex-5-FXT-1136, Xilinx, San Jose, CA, USA). The same authors also proposed, using the same FPGA, a filtering technique using a realtime co-processor SoC based on an Adaptive Moving Average Dual Mode Kalman Filter (AMADMKF, University of Hyderabad, Andhra Pradesh, India) for a fiber-optics gyroscope signal [39]. The presented results show an excellent performance, but the resources used are the 79%. The different techniques and architectures previously discussed based their operating mode upon the use of a batch of data to process them. This has two consequences: a delay in the system response, which prevents detection of changes in real time, and the use of internal memory to store the required data. These disadvantages make it necessary to develop an algorithm to filter the signal allowing real-time change detection.

The contribution of the present work is the development and implementation of a system consisting in the use of an LPFG-based sensor to measure the joint bent. In particular, the system is used for measure specific joint angles of a finger, such as, 0°, 22.5° y 45°. This short range of measure is enough for connecting to a finger, the proposed wearable sensor with the aim of detecting and reproducing finger motions. First, the use of the RLS algorithm is proposed to filter the signal coming from the LPFG-based sensor by using a sample-by-sample approach; then, a Gaussian Mixture Model (GMM) classifies this signal to identify the finger position. By using this strategy, real-time changes can be detected, so the finger position will be updated constantly. Another contribution of the present work is that the methodology can be implemented easily and effectively in a real-time controller. Further, the proposed system is tested to show the filtering and identification techniques performance.

This article is organized as follows: Section 2 describes the theoretical background of the sensor used and models used for filtering and classifying the acquired signal; in Section 3, the proposed system for performing the detection of joint movement is presented and explained. The methodology used to adapt the equations shown in Section 3 to the real-time controller is explained in Section 4. The results obtained are presented and discussed in Section 5. Section 6 draws some conclusions.

## 2. Theoretical Background

### 2.1. LPFG Sensors

An LPFG is an optical sensor that consists of a periodic spatial variation (among 100 μm and 1000 μm) along the fiber longitudinal axis in the refractive index of an optical fiber. The periodic refractive index modulation couples light from a forward-propagating core-guided mode to forward-propagating cladding-guide modes, near certain resonance wavelengths. The light coupled into the cladding modes eventually attenuates due to the high loss of the cladding modes. As a result, the transmission spectrum of an LPFG has a series of discrete attenuation bands near the resonance wavelengths. These discrete attenuation bands are located according to [24]:

(1) $$\lambda^{m}=(n_{\text{eff}}^{01}-n_{\text{eff}}^{m})\Lambda$$

where $n_{\text{eff}}^{01}$ represents the refractive index of the fundamental guided mode, $n_{\text{eff}}^{m}$ stands for the effective index cladding mode of order *m* coupled to the guide mode by the grating and Λ means the period of the LPFG. Details of the fabrication technique are described in [24,40].

### 2.2. Sensing Principle

**Figure 1.**
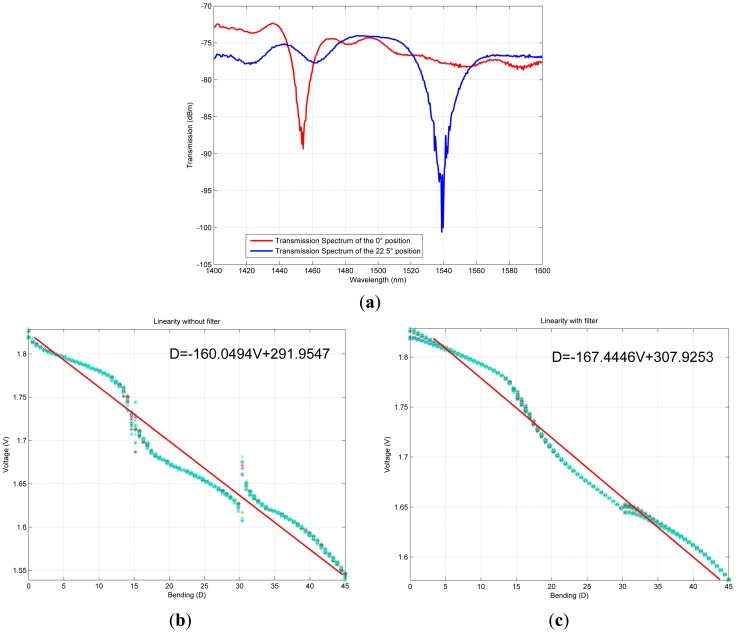
Sensor behavior: (**a**) the response to mechanical action; (**b**) linearity without filter; (**c**) linearity with filter.

Figure 1 illustrates the behavior of the LPFG sensor. Figure 1a shows the attenuation notch in the transmission spectrum for two angles. Note that the LPFG sensor behaves as a selective filter at the wavelength. When mechanical action is applied to the sensor, the frequency response shifts and the amplitude changes, resulting in a variation of the output voltage. This effect establishes a tradeoff between the mechanical action and the sensor response: the light transmitted changes its intensity when the sensor is bent, causing a variable voltage; therefore, a different mean value will be obtained for a different angle, simplifying the angle detection algorithm. Figure 1b illustrates the sensor response to different finger flexions. Every point in the graph is calculated using 20 measurements in ascendant and descendant way, that is, by measuring from an angle of 0 to 45 degrees and *vice versa*. In order to obtain a linear model, the measurement tests were carried out 20 times. As a result of the tests, the equation shown in the chart is obtained, which describes the flexion of the sensor in function of the voltage. The red line represents the best linear fit curve given by the equation. Note the two main inflexion points close to 15 and 30 degrees, and the non-linear behavior of the sensor. However, by filtering the sensor data of Figure 1b, any kind of perturbation or error in the sensor workspace are removed as shown in Figure 1c. That is, the RLS filter (introduced in the next section) reduces the high linearity behavior of the sensor; also, the discontinuity generated at 15 degrees is removed, and the one at 30 degrees is drastically reduced.

As a consequence of the linearity behavior of the sensor, after the filtering stage, a delay is generated during the sensor response. This qualitative comparison of the sensor behavior with and without filter is complemented with the quantitative comparison shown in the results section.

### 2.3. Recursive Least-Square (RLS) Technique

The RLS algorithm is based on the Least-Squares estimation. Assume that **z**(*n*) is the signal measured, which is formed by the intelligent signal **x** (the denoised signal) and a disturbance, *v*(*n*). In addition, the signal **H***^T^*(*n*) is generated from the sensor. Therefore, **z**(*n*) can be expressed as follows (using matrix notation):

(2) $$z(n)=H^{T}(n)x+v(n)$$

The cost function (or the error) is described as:

(3) $$J(\hat{\text{x}})=\sum_{n=1}^{N}{\left\|z(n)-H^{T}(n)\hat{x}\right\|}^{2}$$

where **x̂** is the estimated value. Using the Euclidean matrix norm definition [41], Equation (3) can be expressed as:

(4) $$J(\hat{x})=\frac{1}{2}{\left[z-H\hat{x}\right]}^{T}[z-H\hat{x}]$$

The mathematical operations result in:

(5) $$J(\hat{x})=\frac{1}{2}[z^{T}z-z^{T}H\hat{x}-H^{T}{\hat{x}}^{T}z+H^{T}{\hat{x}}^{T}H\hat{x}]$$

The least-square estimate is defined as the vector that minimizes the cost function, that is:

(6a) $$\frac{\partial J(\hat{x})}{\partial \hat{x}}=-z^{T}H+(H^{T}H){\hat{x}}^{T}=0$$

(6b) $$\hat{x}={\left(H^{T}H\right)}^{-1}H^{T}z$$

Also Equation (6b) can be rewritten as [37]:

(7) $$\hat{x}(N)={\left(\sum_{n=1}^{N}H(n)H{\left(n\right)}^{T}\right)}^{-1}\sum_{n=1}^{N}H(n)z(n)$$

As the filter is implemented in a real-time platform it is necessary to express Equation (7) in a recursive form. From Equation (7), **P**(N) is defined as:

(8) $$P(N)={\left(\sum_{n=1}^{N}H(n)H{\left(n\right)}^{T}\right)}^{-1}$$

**P**^−1^(*N*) can be obtained recursively as the sum of the previous value and an updating factor, as follows:

(9) $$P^{-1}(N)=P^{-1}(N-1)+H(n)H{\left(n\right)}^{T}$$

Next, rewriting Equation (7) by replacing Equations (8) and (9) leads to:

(10a) $$\hat{x}(N)=P(N)\left(\sum_{n=1}^{N-1}H(n)z(n)+H(N)z(N)\right)$$

(10b) $$\hat{x}(N)=P(N)\left(\sum_{n=1}^{N-1}H(n)z(n)+H(N)z(N)\right)$$

(11) $$\hat{x}(N)=\hat{x}(N-1)+P(N)H(N)\left(z(N)+H^{T}(N)\hat{\text{x}}(N-1)\right)$$

The use of Equation (9) requires computing a matrix inversion at each time step, which is known to be slower and more demanding on computer resources. Having considered the matrix inversion lemma, this equation is expressed as:

(12) $$P(N)=P(N-1)-\frac{P(N-1)H(n)H{\left(n\right)}^{T}P(N-1)}{1+H{\left(n\right)}^{T}P(N-1)H(n)}$$

The RLS algorithm is used as a filter, and it is necessary to know the value for the next iteration, **x̂**(*N* + 1) instead of knowing the current, **x̂**(*N*). Thus, the equation for the next value derives from a formally recursive way:

(13) $$\hat{\text{x}}(N+1)=\hat{x}(N)+P(N+1)H(N+1)\left(z(N+1)+H^{T}(N+1)\hat{x}(N)\right)$$

As can be seen in Equation (13), **P**(*N* + 1) is required. Using the recursive way, this value is obtained as:

(14) $$P(N+1)=P(N)-\frac{P(N)H(n+1)H{\left(n+1\right)}^{T}P(N)}{1+H{\left(n+1\right)}^{T}P(N)H(n+1)}$$

### 2.4. Gaussian Mixture Models (GMM)

The GMM are defined as the sum of *K* Gaussian densities [42]:

(15) $$p(\vec{x}|\lambda )=\sum_{i=1}^{K}p_{i}b_{i}(\vec{x})$$

where *x→* is the *D*-dimensional input vector, *b_i_*(*x→*)(*i*=*1, …, K*) is the component density, *p_i_* (*i* = *1*, …, *K*) represents the mixture weights, and *λ* represents the parameters for every class (in this study, an angle). These parameters are defined as:

$$\lambda =\left\{p_{i},{\vec{\mu }}_{i},\Sigma_{i}\right\}\;i=1,\ldots ,M$$

where *μ→_i_* is the mean vector and $\underset{i}{\Sigma }$ is the covariance matrix.

Each component density is a Gaussian function with the form:

(16) $$b_{i}(\vec{x})=\frac{1}{{\left(2\pi \right)}^{D/2}{\left|\underset{i}{\Sigma }\right|}^{1/2}}\exp\left(-\frac{1}{2}{\left(\vec{x}-{\vec{\mu }}_{i}\right)}^{T}\underset{i}{\Sigma^{-1}}(\vec{x}-{\vec{\mu }}_{i})\right)$$

The objective is to detect the finger position (angle) that has the maximum *a-posteriori* probability for a voltage sequence of values. Mathematically this can be expressed as:

(17) $$\theta =\text{arg}\underset{1\leq k\leq M}{\max}P(\lambda_{k}|X)=\text{arg}\underset{1\leq k\leq M}{\max}\frac{p(X|\lambda_{k})Pr(\lambda_{k})}{p(X)}$$

At this point, Equation (17) can be simplified by assuming both *Pr*(*λ_k_*) and *p*(*X*) are independent for every angle. This leads to:

(18) $$\theta =\text{arg}\underset{1\leq k\leq M}{\max}p(X|\lambda_{k})$$

## 3. The Motion Detection System

**Figure 2.**
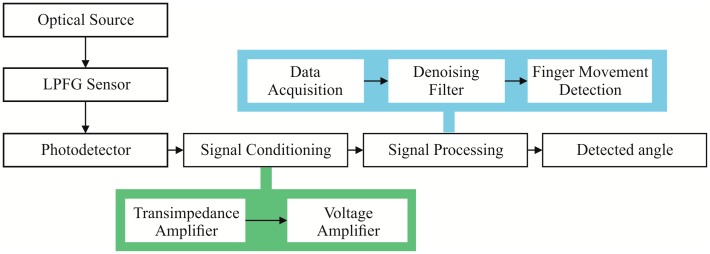
Proposed System block diagram.

The global strategy of the system is depicted in Figure 2. The system uses an LPFG-based sensor, made of a conventional mode fiber (SMF-28) using the technique described in [24], with a grating period (Λ) of 630 μm and a grid length of 12 mm. The sensor is supplied by a Supercontinuum light source (broad spectrum) described in [43]. This type of source generates a spectrum with a high flatness and spectral width of more than 1100 nm (600 nm to over 1700 nm). Then, the sensor is connected to a photodetector to convert from light to an electric current. Rather than working with this electric current, the signal processing stage deals with a voltage signal by performing a current-voltage conversion in the signal conditioning stage. Afterwards, the signal processing stage accomplishes two main tasks: (1) filtering the signal using the RLS algorithm; and (2) classifying the finger position using the GMM technique. In the following subsections, the signal conditioning and processing stages are illustrated in detail.

### 3.1. Signal Conditioning

The photodetector included on the signal conditioning stage works in two different forms: (1) the photodiode mode; or (2) the photovoltaic mode. The former could be the most convenient solution in the proposed system because it delivers a voltage signal, but the response time increases, making it unusable to detect real-time changes. On the other hand, by using the latter, an electrical current signal is delivered, allowing a faster response time due to the reduced junction capacitance. Thus, real-time changes in the measured signal can be detected; therefore, this operation mode has been chosen.

The process involving the conversion from current to voltage signal uses operational amplifiers (op amps) in a configuration known as transimpedance converter. The op amps used in this approach are the LTC1051 [44], which meet the following conditions:

- The signal from an operational amplifier has to be significantly greater than the noise at the output. That is, the offset voltage has to be smaller than the output voltage.
- The noise generated by the op amp has to be sufficiently smaller than the noise generated by the photodetector.
- The noise generated by the feedback resistor (used in a typical transimpedance configuration), which is a type of Johnson-Nyquist noise, has to be sufficiently smaller than the photodetector noise.
- The feedback resistor value has to be chosen carefully in a predefined range, that is, small values produces a small output voltage, on the contrary, large values produces a very noisy signal.

Besides these four conditions, different techniques should be explored to connect the op amps. For the purposes of this article, the auto-zero connection is used. This architecture consists in connecting a two or more stage composite amplifiers structure similar to the chopper-stabilized scheme. The difference between this approach and the chopper architecture is that the stabilizing amplifiers are connected to the main through an additional nulling terminal, instead of connecting to one of the differential inputs; so, high-frequency signals bypass the nulling stage by connecting directly to the main amplifier. Therefore, a stable zero in wide-bandwidth operation is reached, which is one of the desired characteristics in the conditioning stage of the proposed system.

An additional amplifier is required to scale the signal for an optimal use of the Analog-to-Digital Converter (ADC, NI 9215, National Instruments Corporation, Austin, TX, USA). This additional stage involves using an instrumentation op amp (an AD620 model [45]) that produces a high gain on the signal without using more components.

### 3.2. Signal Processing

The conditioned signal is acquired by a 4-channel 16-bit ADC (NI 9215 model [46]), using one channel during the acquisition. Different strategies could be used to filter this signal, using the sample-by-sample approach such as the Kalman filter. If this technique is used, then it is necessary to know an *a-priori* model of the system. On the other hand, the RLS-based estimator performs the filtering task without a predefined model; hence, this approach is more suitable and it is employed in this study.

Once the signal is filtered, a technique to classify the detected voltage is needed. This study considers that it is unnecessary to know which specific finger is bent when a person moves any finger. Therefore, it can be assumed the likelihood for the finger flex fells into a particular notch for each angle allowing the use of a Gaussian distribution. Furthermore, a GMM is an adequate solution for performing the classification.

## 4. Methodology

The proposed system is implemented using the NI cRIO-9024 real-time controller [47] that uses a PowerPC-based microprocessor running at 800 MHz. The aforementioned ADC delivers samples at a rate of 100 kS/s, and it is connected to the controller to filter the signal and compute the joint position. This controller can be configured in two ways: using (1) the Scan Interface; and (2) the LabVIEW FPGA Interface. For the purposes of this study, the first mode is chosen because it allows using the microprocessor as the processor unit. The following subsections describe how Equations (12), (13) and (18) are implemented in the real-time controller.

### 4.1. RLS-Based Filter Implementation

Because the filter is developed in an online way, samples cannot be stored in mass. Saving one sample allows correcting the filter output without compromising the performance, which is the main concern in a real-time system. Therefore, Equations (12) and (13) should be considered in a scalar form.

Assuming that the noise impact is constant, the term **P**(*N* + 1)**H**(*N* + 1) can be replaced with a static gain factor. Bearing this in mind, Equation (13) can be rewritten as:

(19) $$\hat{x}(N+1)=\hat{x}(N)+\frac{1}{t+1}(z(N+1)-\hat{x}(N))$$

where *x̂*(*N* + 1) is the filtered value, *x̂*(*N*) is the previous filtered value, $\frac{1}{t+1}$ is the static gain factor, and *z*(*N* + 1) is the signal from the conditioning stage. It is seen that the gain factor (GF) is an arithmetic division, which is known as a very costly operation in terms of the implementation resources. If the GF is allowed to be only power of 2, then it can be implemented using shift registers, which are less costly and faster than many algorithms used to solve the general division operation.

**Figure 3.**
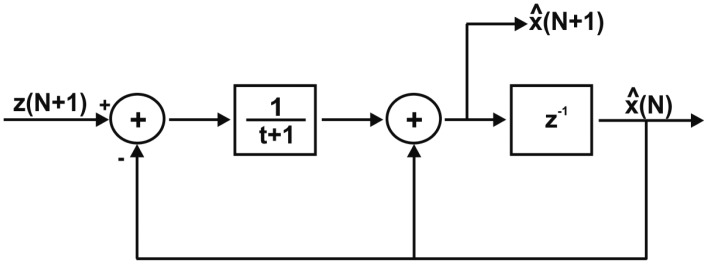
Real-time filter form implementation.

The filter operation can be summarized as follows: once a new sample arrives, its value is arithmetically subtracted with the delay block output value; then, the subtraction result is divided by the gain factor and added to the delayed block output value. The addition result is the filter output. The delay block is also updated since its value is used when a new sample is processed. The aforementioned steps were developed using LabVIEW basic functions. The block diagram that shows how Equation (19) is implemented on the real-time controller is depicted in Figure 3.

### 4.2. GMM Model Implementation

**Figure 4.**
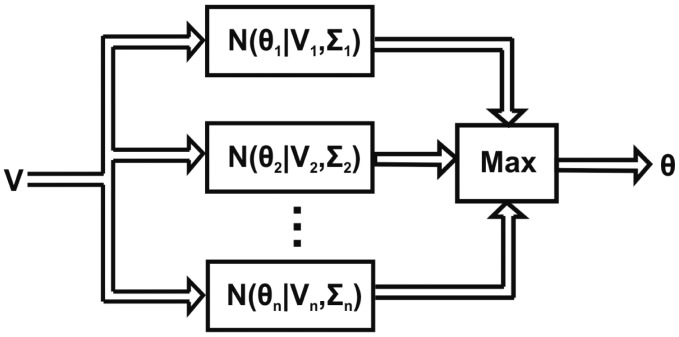
GMM implementation.

Conceptually, the GMM model consists in calculating a finite number of Gaussian functions with their corresponding parameters and maximizing their results to get the most likely coincidence. Therefore, no further modification needs to be done to Equation (18). In the LabVIEW environment these concepts are developed as follows: for every angle, its mean and standard deviation are calculated and arithmetically divided. Once these values are obtained, when a sample arrives, its likelihood for every angle is calculated using the exponential function. Once the probability values are obtained, they are sorted from the highest to the lowest value, being the highest the detected angle. In Figure 4 is shown the block diagram of the implementation.

## 5. Results

**Figure 5.**
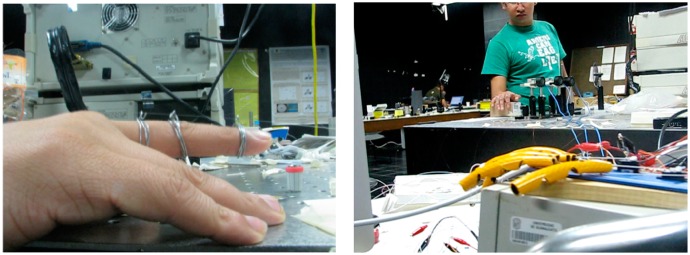
Experimental setup. **Left**: The LFPG sensor adapted to one finger; **Right**: First plane shows the handmade fingers connected to the LFPG sensor in a human hand.

To detect how bent the joint is, movements from 0, 22.5 and 45 degrees are tested to obtain a set of training data. After that, they are processed with the RLS-based filter and the GMM model is created with these values to classify the detected movement. Figure 5 illustrates the LFPG sensor connected to finger joints for monitoring motions, which will be reproduced for handmade improvised fingers. For now, the sensor is only attached to the fingers of the subject, however a wearable version of the sensor could be developed depending on the particular application. The experiment description and a comparative analysis with other proposed sensors are presented and discussed in this section.

### 5.1. Experiment Description

In a sheet, using a protractor, several marks are made to identify the angles to be detected. After that, the sensor is located at every drawn position to get a frame of data (1000 samples). This procedure is repeated 10 times. Then, for every angle, its batch of data is filtered and the required parameters (mean and standard deviation) are obtained to build the GMM model. Once the GMM model is created, the procedure is repeated again 10 times to test the model and get the detection rate.

### 5.2. RLS-Based Filter

**Figure 6.**
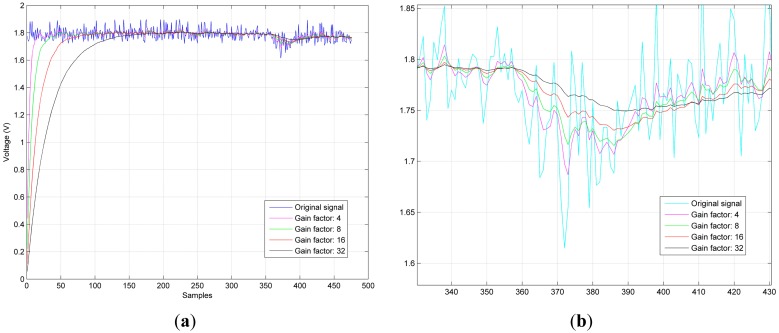
GF effects on (**a**) the filter lag and (**b**) the filter tracking.

Figure 6 shows the Gain Factor (GF) effects during the input signal filtering.

In Figure 6a, the magenta trace represents a GF of 4, the green one a GF of 8, the red one a GF of 16, and the black trace a GF of 32. Notice that, when the GF is modified, an increased delay is produced in the signal tracking and its filtering. Furthermore, the bigger the GF is, the more insensible the filter is to the signal variations (as shown in Figure 6b, a zoomed version of Figure 6a), since the filter will smooth the signal, causing a slowly angle detection as more samples will be need in order to detect the angle. Therefore, the GF size depends on how insensitive the filter needs to behave, and how many samples can be lost without losing knowledge.

### 5.3. GMM Model

**Figure 7.**
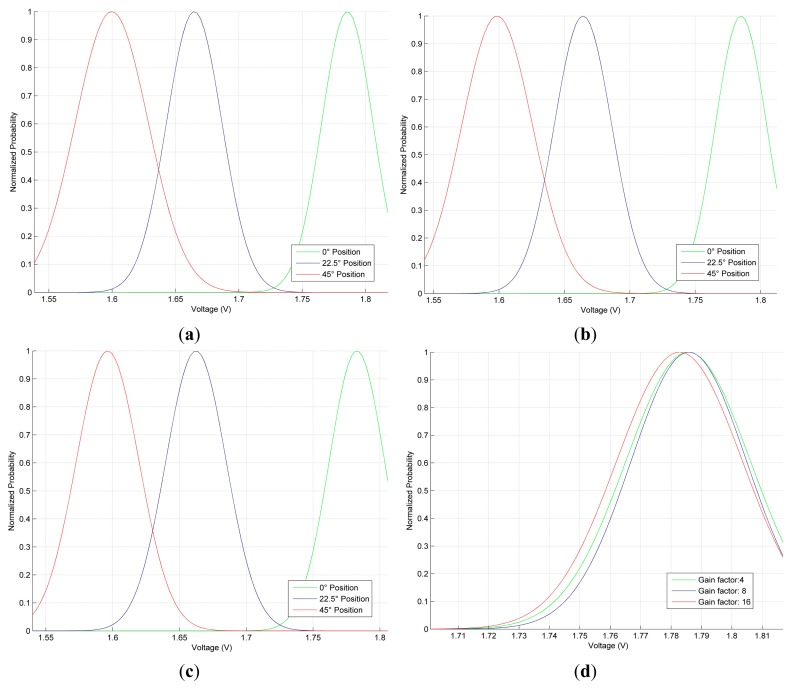
GF Effects in the GMM. (**a**) GF = 4 (**b**) GF = 8 (**c**) GF = 16 (**d**) GF Effects on the GMM of 0 degree.

Several tests performed on the GMM model allow describing some difficulties arising from the analysis of different angle positions. By examining Figure 7a–c, an overlap between certain angles (especially for 0 and 22.5 degrees) can be seen; however, GF helps to overcome this issue. For instance, if the GF is increased the overlapping is reduced, but a carefully selection must be done in order to avoid failures in the rate detection due to the shift in the mean value (see Figure 7d).

The GF size causes a displacement on the Gaussian signals, as a direct function of the GF value; that is, the bigger the factor is, the more displacement occurs, which cause a modification in the original information. This is illustrated on Figure 7d, where the green trace illustrates the Gaussian signal for the 0 degree by using a GF of 4; the blue one shows the Gaussian signal obtained by using a GF of 8. Lastly, the red one shows the Gaussian signal for a GF of 16. The GF influence can be summarized as a progressive displacement and narrowing of the Gaussian signal as the GF becomes bigger. This is an undesirable effect because the Gaussian signals are going to be located one behind the other, which can lead to wrongly detected or undetected movements, since the mean values change depending on the GF value used.

At this point, the effect of the GF can be explained as follows: when selecting a low gain, a wrong operation of the filter is likely to happen as the filter does not perform any smoothing on the signal, causing a misdetection of the detected angle. On the other hand, a larger one would cause a distortion of the original information, since the filter will be less sensitive to the variations of the signal, leading, in the best scenario, to a slower angle detection. For this kind of application, the GF can be located between a magnitude of 4 and 8. In this study, the size selected is 4. The results obtained for the finger-movement detection are presented in the following subsections.

### 5.4. GMM Model Performance

Table 1 shows the finger-movement detection results averaging the 10 tests performed. In this, the first column is the angle to be detected, the first row indicates the detected angle. As can be seen, the GMM model shows that the movement measurement of a joint is possible, with a rate of classification of up to 97%. The best result is obtained when the joint is located at 0 degrees, due to the absence of any evident overlapping with other angles. On the other hand, the worst classification case happens when the angle is 22.5 degrees. This is caused because of the aforementioned overlapping; yet, the detection rate is over 85% in all cases, validating the assumption of a Gaussian distribution for the joint movement. Furthermore, it is also clear the effectiveness by using the RLS-based filter and the selected GF size, since 15 samples are required to obtain the filtered value.

As previously discussed in Section 1, other studies have reported applications that measure when a finger is moved (references [21,22]). Contrary to the aforementioned studies, this study reports how much the finger is moved and the corresponding classification of angle rates. Nevertheless, a quantitative analysis among other similar sensors proposed in the literature [15,33,48] is presented in Table 2. The linear model and seven of the most important features of different sensors are compared: Angle Accuracy, Sensitivity, Standard deviation, Response time, R-square (R2), Adjusted R-square (a-R2) and Root Mean Squared Error (RMSE). The sensors used for the comparison are those proposed by Nishiyama, *et al.* [15] (labeled as Sensor 1), Ferreira da Silva, *et al.* [33] (Sensor 2) and Silva, *et al.* [48] using 1 and 4 loops (Sensor 3).

In Table 2, the first column shows the attributes used for the comparison, the second column corresponds to our proposed-sensor response without any processing, the third and fourth columns show the response of our sensor after the RLS filter with the GF of 4 and 8, respectively. Note that the filtering technique improves the linearity of the sensor and reduces the standard deviation with respect to the sensor raw measurements. One might think that a GF of 8 should be used in order to improve the linearity and the RMSE value; however, the displacement effect in the GMM model (shown in Figure 7d) has to be considered, being this issue the reason for using the GF of 4. On the other hand, the decrease of the standard deviation improves the sensor repeatability and the R2 value and a-R2. Furthermore, the RMSE decrease which means that our sensor, after the filter stage, throws better measurements closer to the real values. However, these improvements imply an increase in the sensitivity and a higher Response time of measurements of the sensor.

The fifth column of the Table 2 shows the quantitative behavior of Sensor 1 [15]; we point out that the sensibility and the standard deviation of our proposed sensor are better (therefore, the repeatability is better), although Sensor 1 is more accurate. The sixth column shows Sensor 2 [33]. As we mentioned above, our proposed sensor without the RLS filter is less accurate, then Sensor 2 is better; however, after the RLS filter, our sensor improves the Sensor 2 accuracy and the response time of measurements. It is important to point out that, the linear model of Sensor 2 is much better than that of our proposed sensor, due to the high value of a-R2.

Finally, the seventh and eight columns show two different sensors proposed by Silva *et al.* [48]. The authors explain that, depending on the number of loops in the sensors, these display different behaviors: the more loops the sensor has, the better the sensor response. Even if these sensors show good values for sensibility and R2, the response time of measurements provided is bigger than that of our proposed sensor, limiting Sensor 3's applications. Regarding the response time presented in the aforementioned table, the information shown is taken from the sampling rate, as some reported sensors operate in real-time. In a real-time FPGA-based system, any change in the measured variable is detected in the next sample, which is obtained at the sampling frequency. Thus, the response time for real-time systems is the sampling rate. In general, the proposed sensor has good qualities and trade-offs among all the exposed features for a real time application.

## 6. Conclusions

This work proposes a new technique to detect how much a finger is flexed using a LPFG-based sensor. The overall methodology consists in the use of the RLS algorithm to filter the signal and the GMM model to classify the detected movement. By taking advantage of the LPFG sensor manufacture by electric arc discharge, this is a low-cost system. However, due to this manufacturing process, the response for each sensor is different, and thus the probabilistic model GMM has been used to account for this effect. The GMM model is often easy to tune; therefore, the proposed strategy is easily adapted to different sensor responses derived from sensor manufacturing process differences. Even though the GMM model shows certain immunity to noise, a filtering stage using the RLS algorithm is required to improve the noise-to-signal ratio. The results illustrate that the proposed methodology is capable of quantifying the finger movement; in the worst case scenario the technique effectiveness is in excess of 85%. This algorithm can be further improved by using more than one Gaussian for every angle and a GF self-adjustment in the RLS-based filter. No comparison can be established with other studies as none of them report detection rates for flexing joints.

For high speed processing, an FPGA implementation providing the real time rate required in any actual system is needed. Moreover, the proposed filtering method can be straightforwardly implemented on the FPGA without any previous adjustment analysis. In addition, this work can be used for further research in a sophisticated mechatronic hand. For instance, a dynamic motion of the finger can be considered to increase the classification rate by rejecting the outliers not expected in the predefined finger motion.

## Figures and Tables

**Table 1.** Confusion matrix of the detected angles using the GMM model.

**Angle**	**0°**	**22.5°**	**45°**
0°	97%	3%	0%
22.5°	0.8%	86.6%	12.6%
45°	0%	10.4%	89.4%

**Table 2.** Analysis comparative of seven sensor features and the lineal model for the proposed sensor and three more sensors proposed in the literature (--- means that the corresponding data is not provided by the authors).

**Feature**	**Sensor without Filter**	**Sensor with Filter (GF = 4)**	**Sensor with Filter (GF = 8)**	**Sensor 1 [15]**	**Sensor 2 [33]**	**Sensor 3 [48]–1 Loop**	**Sensor 3 [48]–4 Loop**
Accuracy of Angle [deg.]	2.2925	1.9587	1.6692	0.6800	2.0000	---	---
Sensitivity	0.8422 V	0.8470 V	0.8527 V	0.9400 dB	---	0.1970 dBm	0.6400 dBm
Standard Deviation [%]	0.2584	0.1600	0.1476	0.7000	---	0.80	1.00
Measurements Response Time	10 μs	100 μs	100 μs	---	31 ms–500 μs	∼100 ms	∼100 ms
Real Time Processing	Yes	Yes	Yes	Yes	Yes	Yes	Yes
R-Square (R2)	0.9639	0.9710	0.9804	---	---	0.9630	0.9890
Adjusted R-Square (a-R2)	0.9635	0.9706	0.9801	---	0.9993	---	---
Root Mean Squared Error (RMSE)	2.5288	2.2694	1.8651	---	---	---	---
